# An assessment of quality of life for early phase after adjuvant radiotherapy in breast cancer survivors: a Korean multicenter survey (KROG 14–09)

**DOI:** 10.1186/s12955-017-0673-1

**Published:** 2017-05-10

**Authors:** Chai Hong Rim, Sung-Ja Ahn, Jin Hee Kim, Won Sup Yoon, Mison Chun, Dae Sik Yang, Jong-Hoon Lee, Kyubo Kim, Moonkyoo Kong, Suzy Kim, Juree Kim, Kyung Ran Park, Young-Joo Shin, Sun Young Ma, Bae-Kwon Jeong, Su Ssan Kim, Yong Bae Kim, Dong Soo Lee, Jaehyung Cha

**Affiliations:** 1 Department of Radiation Oncology, Yonsei Cancer Center, Yonsei University College of Medicine, Seoul, Republic of Korea; 2 Department of Radiation Oncology, Chonnam National Univiersity Hwasun Hospital, Hwasun, Jeollanam-do Republic of Korea; 3 Department of Radiation Oncology, Dongsan Medical Center, Keimyung University, Daegu, Republic of Korea; 4 Department of Radiation Oncology, Ansan Hospital, Korea University, 123 Jeokgeum-ro, Danwon-gu, Ansan, Gyeonggi-do 15355 Republic of Korea; 5 Department of Radiation Oncology, Ajou University, Suwon, Gyeonggi-do Republic of Korea; 6 Department of Radiation Oncology, Guro Hospital, Korea University, Seoul, Republic of Korea; 7 Department of Radiation Oncology, St. Vincent’s Hospital, College of Medicine, The Catholic University of Korea, Seoul, Republic of Korea; 8 Department of Radiation Oncology, Seoul National University Hospital, Seoul, Republic of Korea; 9 Department of Radiation Oncology, Mokdong Hospital, Ewha Woman’s University School of Medicine, Seoul, Republic of Korea; 10 Department of Radiation Oncology, Kyung Hee University Medical Center, Kyung Hee University School of Medicine, Seoul, Republic of Korea; 11 Department of Radiation Oncology, Boramae Medical Center, Seoul National University, Seoul, Republic of Korea; 12 Department of Radiation Oncology, Cheil General Hospital and Women’s Healthcare Center, Dankook University College of Medicine, Seoul, Republic of Korea; 13 Department of Radiation Oncology, Sanggye Paik Hospital, Inje University, Seoul, Republic of Korea; 14 Department of Radiation Oncology, Kosin University Gospel Hospital, Busan, Republic of Korea; 15 Radiation Oncology, Gyeongsang National University School of Medicine and Gyeongsang National Univeristy Hospital, Jinju, Gyeongsangnam-do Republic of Korea; 16 Department of Radiation Oncology, Asan Medical Center, University of Ulsan College of Medicine, Seoul, Republic of Korea; 17 Department of Radiation Oncology, Uijeongbu St. Mary’s Hospital, The Catholic University of Korea, Uijeongbu, Gyeonggi-do Republic of Korea; 18 Department of Medical Science Research Center, Ansan Hospital, Korea University, Ansan, Gyeonggi-do Republic of Korea

**Keywords:** Breast neoplasm, Quality of life, Radiation therapy, EQ-5D, EORTC-QLQ-BR23

## Abstract

**Backgrounds:**

Quality of life (QoL) has become a major concern as the survival time of breast cancer increases. We investigated the changes in QoL through comprehensive categorical analysis, for the first three years after breast cancer treatment including radiotherapy.

**Methods:**

A total of 1156 patients were enrolled from 17 institutions. All survivors were grouped according to a surveillance period of 9–15 months (first year), 21–27 months (second year), and 33–39 months (third year) from the end of radiotherapy. The 5-dimensional questionnaire by the EuroQol group (EQ-5D) and the EORTC Quality of Life Questionnaire; breast cancer specific module (QLQ-BR23) were checked by self-administrated method.

**Results:**

First, second and third year groups comprised 51.0, 28.9, and 21.0%. In EQ-5D-3 L (3-Likert scale) analysis, pain/discomfort and anxiety/depression categories showed lower QoL. In multivariate analyses of EQ-5D-VAS (visual-analogue scale), categories of pain/discomfort and self-care were improved with time; axillary dissection was a significant clinical factor deteriorates pain/discomfort, self-care and usual activities. In QLQ-BR23 analysis, the lowest scored category was sexual activity, followed by sexual enjoyment, future perspective, and hair loss, and the best scored category was breast symptoms. In multivariate analyses, arm symptoms, breast symptoms and body image were improved with time.

**Conclusions:**

Categories of pain/discomfort and self-care in EQ-5D-VAS, arm/breast symptoms and body image in QLQ-BR23 were improved, while categories of anxiety/depression and future perspective BR23 were not, suggesting necessity of psychosocial support. This research provides comprehensive information on the categorical aspects of QoL and changes during early follow-up after breast cancer treatment.

## Backgrounds

Breast cancer is the most frequently diagnosed cancer among females worldwide, and accounts for 25% of all cancer cases [1]. After a continuous increase during the 1980’s to 1990’s, the incidence rates of Western countries in early 2000’s declined or were stable, likely because of the reduced use of female hormones or plateaus in participation of mammographic screening. Death rates have been declining due to early detection or improved treatment [2]. In South Korea, similar tendencies of incidence and mortality were observed. The incidence has been continuously rising by 5.6% per year from 1999 to 2013, and the 5-year survival rate was 91.5% between 2009 and 2013 comparing the 5-year survival rate of 77.9% between 1993 and 1995 [3].

Since there have been significant increases in the survival of breast cancer patients, there are emerging issues concerning quality of life (QoL) after surgery and adjuvant treatment [4]. Besides the progress of surgical methods, chemotherapy and hormone therapy, adjuvant radiotherapy plays an important role to improve overall survival. Recent long-term follow-up outcomes re-confirmed the survival gains of adjuvant radiotherapy [5, 6].

Previous studies revealed that breast cancer survivors continue to experience adverse effect of cancer itself and treatment years after the treatment finishes, including fatigue, pain, fear of recurrence, depressive symptoms, and sexual discomfort. All those symptoms become a hindrance to the recovery of patients’ daily activities or self-management [7, 8].

However, most data about QoL of breast cancer patients derived from cross-sectional studies, which are not able to indicate the course of QoL over time. Some longitudinal studies about QoL of breast cancer patients might have the drawbacks of a small study population with a limited statistical power or outdated version to present recent therapeutic principle [7, 8]. Additionally, there are very few studies that are based on the general QoL after adjuvant radiotherapy.

As the concern regarding the QoL of cancer survivors is rising, we designed a nationwide multi-institutional study to examine the QoL of breast cancer survivors, in the critical early phase up to 3 years after a completion of adjuvant radiotherapy.

## Methods

### Study population recruitment

The population of our study consisted of patients diagnosed with breast cancer, treated with definite surgery followed by adjuvant radiotherapy, regardless of any other adjuvant. We performed a questionnaire survey to patients who visited the outpatient clinic and corresponded to the periods of 3 months before and after 1, 2, and 3 years after completion of radiotherapy. For example, the population in the 1st year group visited from 9 to 15 months after completion of radiotherapy. The included patients in our study were who; 1) successfully completed planned radiotherapy; 2) were aged between 20 and 70 at the initiation of radiotherapy; 3) were able to communicate in the Korean language and 4) agreed to answer our questionnaire. Exclusion criteria were those who; 1) had any locoregional recurrence or metastasis during or after radiotherapy; 2) had a history of any malignancy except non-melanoma skin cancer; 3) had a disability below Eastern Cooperative Oncology Group performance scale 2 due to physical problem other than breast cancer; and 4) is male.

The population size of our study was estimated using our country and United States cancer statistics. In the year 2011 in South Korea, the newly diagnosed breast cancer patients totaled 15,356, and 2063 people died due to breast cancer. Based on the above values, we assumed that about 13,000 breast cancer patients survived each year [9]. According to the Surveillance, Epidemiology, and End Results Program data of United States, 51% patients with early breast cancer (stage I or II) and 44% of advanced breast cancer (stage III or IV) received adjuvant radiotherapy [10]. Considering this, we expect that about 50% of breast cancer patients would receive adjuvant radiotherapy in South Korea, and we presumed that the number was about 6500 patients each year. Regarding that our exclusion criteria may exclude about 500 patients in each year group, we can estimate that the parent population would be about 6000 patients in a year. With this estimation and the following formula, we calculated the size of sample for our study.

$$ n={{z^2}_{a/2}}^{\ast }\ {0.5}^2/\left\{{\left( Sampling\  error\right)}^2+{{z^2}_{a/2}}^{\ast }\ {0.5}^2/ N\right\} $$

(Confidence level 90% z_a/2_ = 1.65, Confidence level 95% z_a/2_ = 1.96, Confidence level 99% z_a/2_ = 2.54, N = parent population).

As we used a confidence level of 95% and sampling error of +/−5%, we calculated that 384 patients was the sample size needed per year. Consequently, the ideal size of sample for 3 years is 1152. Hence, our goal was to perform our survey to about 1100 patients. However, due to the characteristic of clinical follow-up that the rate of regular checkup is declining as time passes, we applied sample errors and set the sample sizes as followed; 1) 317–474 survivors at the 1st year with +/−4.5–5.5% sample error; 2) 227–317 survivors at the 2nd year with +/−5.5–6.5% sample error; and 3) 170–227 survivors at the 3rd year with +/−6.5–7.5% sample error.

### QoL measurement

The QoL of study participants was assessed using the 5-dimensional questionnaire by EuroQol group (EQ-5D) to evaluate general health-related QoL and the European Organization for Research and Treatment of Cancer Quality of Life Questionnaire–breast cancer specific module (EORTC QLQ-BR23) to evaluate characteristic QoL of breast cancer patients.

The EQ-5D, a validated questionnaire translated into Korean, [11] is a widely-used questionnaire to evaluate general health status in 5 categories (mobility, self-care, usual activities, pain/discomfort, and anxiety/depression). Each category was assessed by 3-grade Likert scale: 1, No problem; 2, some or moderate problems; 3, extreme problems (EQ-5D-3 L). In addition, each category was also assessed on a visual analogue scale (VAS) where the endpoints are classified as follows: ‘the best imaginable health state’ =100 and the ‘worst imaginable health state’ =0 (EQ-5D-VAS).

The EORTC QLQ-BR23 instrument comprised of 23 questions, which can be divided into 8 categories and 2 areas which are functional (body image, future perspective, and sexual activities and enjoyment) and symptomatic (arm symptom, breast symptoms, side effects of systematic therapy, and upset by hair loss). Each question is scored on a 4-point Likert scale from 1 (not at all) to 4 (very much), and we arranged the data to the above-mentioned categorical basis and linearly transformed them into scores from 0 to 100; a higher score represented better satisfaction with their QoL. The Korean version of EORTC QLQ-BR23 has been tested in a previous study and was shown to be a valid measurement to assess QoL of breast cancer patients [12].

### Clinical and sociodemographic factors acquirement

A researcher in each institution filled out the clinical factors, such as the time of surveillance, type of breast surgery, type of axillary surgery (axillary dissection, sentinel lymph node dissection, or none), chemotherapy, hormone therapy, target therapy, and the extent of radiotherapy. The sociodemographic factors and QoL were acquired by the self-administration method from patients. As for personal and sociodemographic factors, age, body mass index, menopausal status before diagnosis of breast cancer, periodic medication (cardiovascular, diabetic, and psychotropic drug), employment status, household income, education status, hobbies, alcohol consumption, smoking, and use of health supplement were considered.

In addition, the questionnaire of Global physical activity question (GPAQ) of the World Health Organization (WHO) to assess physical activeness was performed. The GPAQ was developed by the WHO for physical activity surveillance in many countries. It collects information on physical activity participation in activity at work, travel to and from places, and recreational activities. The questionnaire is comprised of questions asking about the number of days when a certain degree of activity is performed, and the actual time spent for the activity during the day, for last 7 days. The GPAQ was assorted into 3 grades according to total time of activity, which is calculated by multiplying the ‘number of days with certain activity per week’ and ‘actual time of activity in the day’.

The numerical values calculated through the above method were used for QoL analysis as a variable named physical activity. The GPAQ has been validated and applied in diverse studies worldwide including Asian countries [13, 14].

### Data assessment and statistical methods

We assorted our study participants into 3 groups according to their surveillance period after the end of radiotherapy, which were the 1st year group (9–15 months), the 2nd year group (21–27 months), and the 3rd year group (33–39 months) from the end of radiotherapy. A chi-square test was performed to evaluate distribution of clinical and sociodemographic variables according to surveillance period.

For missing value control, we regarded clinical factors as missing value that chemotherapy less than 3 cycles, target therapy or hormone therapy less than 6 months. If each category of EQ-5D-VAS is only omitted with written EQ-5D-3 L, the omitted EQ-5D-VAS was replaced according to the median VAS for same grade of each category. In GPAQ, if our participants answered as ‘I do not know/I am not sure’ to the question asking actual time of activities, we replaced with median value of responders.

To identify which category of QoL is mostly deteriorated after breast cancer treatment, Cochran–Mantel–Haenszel analysis to assess the EQ-5D-3 L and multiple comparison test after Kruskal-Wallis analysis to assess EQ-5D-VAS and EORTC QLQ-BR23 were conducted.

For univariate analysis, Chi-square test or Cochran-Mantel-Haenszel analysis was performed to investigate the relationship between various factors including the surveillance period, clinical and sociodemographic factors, and physical activity and EQ-5D-3 L.For EQ-5D-VAS and EORTC QLQ-BR23, Kruskal-Wallis analysis was performed. Multiple regression analysis using step-wise methods were performed for the factors found to have a *p* value <0.1 in univariate analysis for EQ-5D-VAS and EORTC QLQ-BR23.All analyses were executed using the IBM SPSS statistics20 (IBM Inc., NY, USA) and the R version 3.2.0 (The R Foundation).

## Results

### Characteristics of participants

From August 2014 to September 2015, 1156 women from 17 hospitals consented and answered questionnaire. With regard to the surveillance period, 587 (58.1%), 332 (30.7%) and 231 (11.2%) patients were corresponded to the first, second and third year group, respectively. The sampling errors with 95% confidence level of the first, second and third year groups were estimated as 3.8, 5.2 and 6.3%, respectively. The surveillance period was missed in 6 questionnaires.

The clinical characteristics of study participants are shown in Table 1. Breast conserving surgery was performed in 91.9% of cases and patients with advanced stage of III or higher were in 11.4% of cases. Radiation field included the regional lymph nodes in 75% of cases and boost to tumor bed was performed in 89.7% of cases. Median dose to whole breast/chest wall and boost was 50.4 Gy and 10.0 Gy, respectively. Assorted by surveillance period, there was no statistically significant difference except for the variables of age and hormone therapy. The difference of age distribution may be reflected by the character of our study and as the surveillance period increases, the median age (50.0, 51.8 and 52.1 years at first, second and third year group, respectively) increases. Regarding hormone therapy, we have more study participants who did not received hormone therapy in the first year group than other groups (29.1 vs. 19.5 vs. 18.6) since some patients in first year group had received hormone therapy for less than 6 months and were considered as missing values. The sociodemographic status was assorted by the surveillance period and is represented in Table 2. There is no statistically significant difference in distribution except variables including hobbies. Regarding variables of hobbies, there are more participants who were categorized as ‘actively participating’ in the third year group.

**Table 1:** Clinical characteristics of survivors

		1st year group	2nd year group	3rd year group	
		*N* (%)	*N* (%)	*N* (%)	^*p*^ value^a)^
Age (years)	Median	50.0	51.8	52.1	0.010
	<41	63 (10.8)	23 (7.0)	15 (6.5)	
	41–50	255 (43.8)	126 (38.2)	88 (38.3)	
	51–60	205 (35.2)	132 (40.0)	87 (37.8)	
	>60	59 (10.1)	49 (14.8)	40 (17.4)	
Stage of disease					0.105
	0	86 (14.7)	37 (11.2)	28 (12.1)	
	I	250 (42.8)	156 (47.4)	107 (46.3)	
	II	190 (32.5)	101 (30.6)	60 (26.0)	
	III-IV	58 (9.9)	35 (10.6)	36 (15.6)	
Menstrual status					0.383
	Premenopausal	350 (60.1)	195 (59.0)	148 (64.6)	
	Postmenopausal	232 (39.9)	135 (40.9)	81 (35.4)	
Breast surgery					0.630
	Breast-conserving surgery	541 (92.6)	302 (91.2)	210 (90.9)	
	Mastectomy	43 (7.4)	29 (8.8)	21 (9.1)	
Axillary surgery					0.206
	None	62 (10.7)	27 (8.2)	25 (10.9)	
	Sentinel node dissection	293 (50.3)	190 (57.4)	128 (55.9)	
	Axillary node dissection	227 (39.0)	114 (27.3)	76 (33.2)	
Radiotherapy field					0.905
	Breast only	435 (74.4)	251 (75.6)	174 (75.3)	
	Breast and regional nodes	150 (25.6)	81 (24.4)	57 (24.7)	
Chemotherapy					0.469
	No	211 (36.3)	125 (37.8)	75 (32.8)	
	Yes	371 (63.7)	206 (62.2)	154 (67.2)	
Hormone therapy					<0.001
	No	163 (29.1)	64 (19.5)	43 (18.6)	
	Yes	397 (70.9)	265 (80.5)	188 (81.4)	
Target therapy					0.855
	No	507 (88.9)	294 (89.4)	203 (87.9)	
	Yes	63 (11.1)	35 (10.6)	28 (12.1)	

^a)^Pearson’s Chi-square test

**Table 2:** Sociodemographic characteristics of survivors

		1st year group	2nd year group	3rd year group	
		*N* (%)	*N* (%)	*N* (%)	^*p*^ value^a)^
Educational status^b)^					0.238
	Low or middle	350 (61.0)	193 (59.6)	152 (66.4)	
	High	234 (39.0)	131 (40.4)	77 (33.6)	
Any family member living together					0.311
	Yes	511 (89.2)	294 (89.4)	197 (85.7)	
	No	62 (10.8)	35 (10.6)	33 (14.3)	
Change of employment (before ➔ after)					0.807
	Unemployed ➔ Unemployed	251 (42.8)	138 (41.6)	107 (46.3)	
	Employed ➔ Unemployed	123 (21.0)	69 (20.8)	42 (18.2)	
	Employed/Unemployed ➔ Employed	213 (36.3)	125 (37.7)	82 (35.5)	
Household income ($/month)^c)^					0.594
	< 2500	263 (46.8)	133 (41.8)	105 (46.5)	
	2500–4000	202 (35.9)	124 (39.0)	77 (34.1)	
	> 4000	97 (17.3)	61 (19.2)	44 (19.5)	
Hobbies					0.013
	Very active	99 (17.1)	74 (22.5)	64 (27.8)	
	Moderate	349 (60.3)	180 (54.7)	120 (52.2)	
	Rare	131 (22.6)	75 (22.8)	46 (20.0)	
Smoking status (Current)					0.116
	Non-smoker	575 (98.3)	317 (96.1)	223 (97.0)	
	Smoker	10 (1.7)	13 (3.9)	7 (3.0)	
Alcohol consumption					0.128
	No	455 (77.6)	265 (80.3)	168 (73.0)	
	Yes	131 (22.4)	65 (19.7)	62 (27.0)	
Physical activity					0.596
	High	183 (33.8)	106 (33.0)	64 (29.0)	
	Moderate	188 (34.2)	111 (34.6)	74 (33.5)	
	Low	176 (32.0)	104 (32.4)	83 (37.6)	

^a)^Pearson’s Chi-square test

^b)^Low or Middle = High school graduated or below, High = College graduated or higher

^c)^Household income is estimated to United State dollar from Korean won, with exchange rate of 1 USD for 1200 KRW. The cut-off values are one-third and two-third points according to Korean Statistics

### EQ-5D-3 L

The answer rate of grade 2 or 3 was highest in the pain/discomfort category, followed by anxiety/depression (Table 3). The grade 2 answer for the pain/discomfort category was significantly higher than expected. With a lapse of time, usual activity (*p* = 0.038) and pain/discomfort (*p* < 0.001) categories were improved, but the other categories showed no significant change. The trend that pain/discomfort grade 2 is measured as higher than expected, persisted in all surveillance periods. We summate grades of all 5 categories into total score ranged from 5 to 15, to reflect general QoL. The total score showed significant improvement with lapse of time (*p* = 0.001).

**Table 3:** Result of the EQ-5D-3 L

	Total			1st year group			2nd year group			3rd year group		
EQ-5D grade^a)^	1	2	3	1	2	3	1	2	3	1	2	3
	*N* (%)	*N* (%)	*N* (%)	*N* (%)	*N* (%)	*N* (%)	*N* (%)	*N* (%)	*N* (%)	*N* (%)	*N* (%)	*N* (%)
Mobility	1009 (88.6)	127 (11.2)	3 (0.3)	517 (88.8)	64 (11.0)	1 (0.2)	289 (87.8)	38 (11.6)	2 (0.6)	203 (89.0)	25 (11.0)	0
Self-care	1101 (96.7)	33 (2.9)	4 (0.4)	562 (96.6)	19 (3.3)	1 (0.2)	318 (97.0)	7 (2.1)	3 (0.9)	221 (96.9)	7 (3.1)	0
Usual activities	984 (86.4)	153 (13.4)	2 (0.2)	492 (84.5)	89 (15.3)	1 (0.2)	286 (86.9)	43 (13.1)	0	206 (90.4)	21 (9.2)	1 (0.4)
Pain/Discomfort	552 (48.5)	574 (50.4)	12 (1.1)	248 (42.6)	328 (56.4)	6 (1.0)	166 (50.6)	159 (48.5)	3 (0.9)	138 (60.5)	87 (38.2)	3 (1.3)
Anxiety/Depression	655 (57.6)	470 (41.3)	13 (1.1)	324 (55.7)	254 (43.6)	4 (0.7)	189 (57.6)	135 (41.2)	4 (1.2)	142 (62.3)	81 (35.5)	5 (2.2)
*p* value^b)^			<0.001			<0.001			<0.001			<0.001

*Abbreviation*: *EQ-5D 3 L* EuroQol group 5-dimensional 3-Likert scale questionairre

^a)^Grade 1 = No problem, Grade 2 = Some/moderate problem, Grade 3 = Extreme problem

^b)^Cochran-Mantel-Haenszel analysis

### EQ-5D vas

**Fig. 1:**
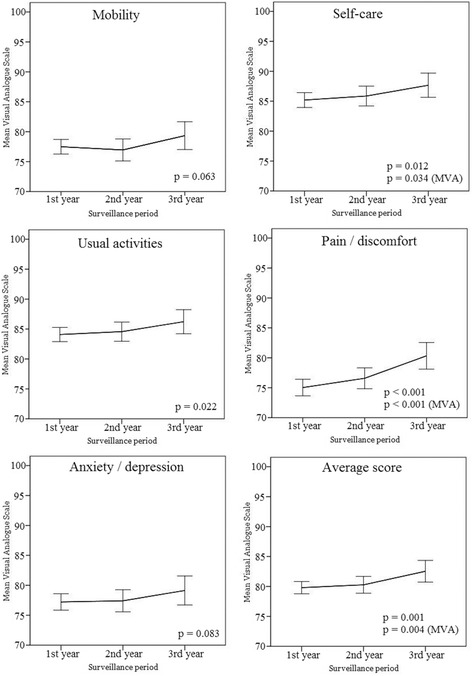
Longitudinal comparison of EQ-5D VAS scores according to surveillance period. Higher scores represented better satisfaction with their quality of life. The 95% confidence intervals are represented at the each point of surveillance period. Statistically significant *p*-values through multivariate analysis (comparing 1st year and 3rd year) are shown. Abbreviations: MVA, multivariate analysis

The EQ-5D VAS showed that pain/discomfort, anxiety/depression, and mobility categories were significantly lower than usual activity and self-care categories. The same trend was observed in all surveillance periods. The categories of pain/discomfort (*p* < 0.001), self-care (*p* = 0.012) and usual activity (*p* = 0.022) were improved with a lapse of time (Fig. 1). We calculated the average scores of 5 categories to indicate general QoL, and the average score was improved with lapse of time (*p* = 0.001).

We performed multivariate analysis to factors showed to be significant in univariate analysis, and the clinical factors, such as stage of disease, surgery type, chemotherapy, hormone therapy, target therapy, and extent of radiotherapy did not affect EQ-5D-VAS significantly (Table 4). The axillary dissection (axillary dissection Vs. none) deteriorated the self-care, usual activities, pain/discomfort, and the average score of EQ-5D-VAS (*p* = 0.039, *p* < 0.001, *p* = 0.004, and *p* = 0.004, respectively). With the lapse of time, the categories of pain/discomfort (*p* < 0.001), self-care (*p* = 0.034) and the average score (*p* = 0.004) were improved in multivariate analyses.

**Table 4:** Multivariate analyses of EQ-5D VAS

	Mobility OR (SE), ^*p*^ value	Self-care OR (SE), ^*p*^ value	Usual activities OR (SE), ^*p*^ value	Pain/Discomfort OR (SE), ^*p*^ value	Anxiety/Depression OR (SE), ^*p*^ value	Average score OR (SE)*,* ^*p*^ value
Disease free period						
(3rd year/1st year)		2.41 (1.13), 0.034		4.48 (1.27), <0.001		2.69 (0.94), 0.004
Axillary dissection						
(axillary/none)		−1.99 (0.96), 0.039	−3.89 (0.93), <0.001	−3.13 (1.09), 0.004		−2.32 (0.80), 0.004
Physical activity						
(high/low)	5.42 (1.19), <0.001		2.42 (0.94), 0.010	2.99 (1.11), 0.007		4.06 (0.94), <0.001
(moderate/low)	4.20 (1.19), <0.001					3.17 (0.93), 0.001
Employment change						
(employed/employed➔Unemployed)	3.07 (1.01), 0.002	2.08 (0.96), 0.031	2.71 (0.92), 0.003		3.92 (1.10), <0.001	2.57 (0.80). 0.001
Educational status						
(high/low or middle)	2.66 (1.07), 0.013	2.74 (1.04), 0.008	2.11 (1.00), 0.34			2.10 (0.86), 0.014
Menopausal status						
(post−/pre-)		−2.24 (1.09), 0.027	−3.67(0.95), <0.001	−3.17 (1.09), 0.004	−2.37 (1.10), 0.031	−2.45 (0.82), 0.003
Household income						
(low/high)	−5.47 (1.05), <0.001	−2.29 (1.00), 0.022	−2.96 (0.97), 0.002	−6.07 (1.44), <0.001	−2.26 (1.07), 0.035	−2.78 (0.83), 0.001
(low/mid)				−4.15 (1.45), 0.004		
Hobbies						
(active/rare)		3.44 (1.12), 0.002	3.38 (1.07), 0.002	3.22 (1.27), 0.011	7.55 (1.57), <0.001	3.71 (0.93), <0.001
(moderate/rare)					2.63 (1.31), 0.045	
Medication of diabetes						
(yes/no)		−5.01 (1.82), 0.005				
Medication of psychotropic drug						
(yes/no)			−3.87 (1.71), 0.24	−6.22 (2.03), 0.002	−11.17 (1.99), <0.001	−5.72 (1.49), <0.001
Alcohol consumption						
(yes/no)					−3.07 (1.26), 0.014	

Negative sign is a negative effect and positive sign is a positive effect

*Abbreviations*: *EQ-5D VAS* EuroQol group 5-dimensional visual analogue scale questionnaire, *OR* odd ratio, *SE* standard error

For sociodemographic factors, menopausal status (*p* = 0.003) before surgery, educational status (*p* = 0.014), household income (*p* = 0.001), employment change (*p* = 0.001), activity of hobbies (*p* < 0.001), physical activity (*p* < 0.001), and psychotropic medication (*p* < 0.001) were significant factors influencing the average score of EQ-5D. Psychotropic medication, employment change and activity of hobbies were found to be strongly related to anxiety/depression with *p* < 0.001. Household income was a strong factor for pain/discomfort and mobility. Physical activity was another strong factor for mobility.

### EORTC QLQ-BR23

**Fig. 2:**
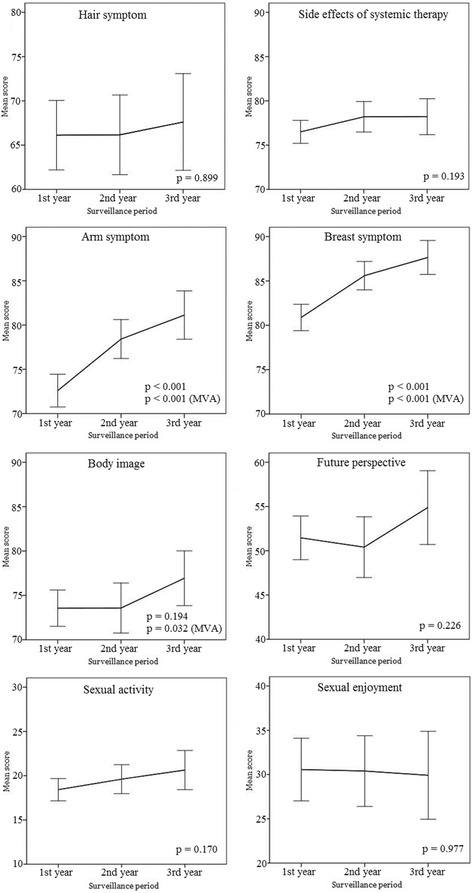
Longitudinal comparison of EORTC BR-23 scores according to surveillance period. Higher scores indicate better health-related quality of life. The 95% confidence intervals are marked at the each point of surveillance period. Statistically significant *p*-values through multivariate analysis (comparing 1st year and 3rd year) are shown. Abbreviations: MVA, multivariate analysis

The lowest scored category was sexual activity, followed by sexual enjoyment, future perspective, hair loss, body image, arm symptoms, side effects of systematic therapy, and breast symptoms. There were no significant differences between scores of body image, arm symptoms, and side effects of systematic therapy, while all the other categorical scores were significantly different between each other. These trends were observed in all surveillance periods. The categories of arm symptoms (*p* < 0.001) and breast symptoms (*p* < 0.001) were improved with time passage. Other categories showed no statistically significant change with time (Fig. 2.).

For upset by hair loss category, the answer rate was relatively low (55.6%) as compared to other categories, since only the participants who experienced actual hair loss answered the question. There were some missing answers for sexual activity (6.1%), and the answer rate of sexual enjoyment category was only 43.9%, which is suggested to be answered from whom had had sexual activity.

In multivariate analysis, the axillary dissection (axillary dissection Vs. none) and stage of disease were significant clinical factors for the symptomatic area (Table 5). For the functional area, the type of breast surgery influenced body image, chemotherapy affected body image and sexual activity, and the axillary dissection (axillary dissection Vs. none) was the factor which affected sexual enjoyment. Various sociodemographic factors strongly affected each QoL category; household income for side effects of systematic therapy; activity of hobbies for side effects of systematic therapy, arm symptoms, future prospective and sexual activity; medication of psychotropic drug for side effects of systematic therapy and body image; employment change for body image and future prospective were observed with strong significance (*p* < 0.001).

**Table 5:** Multivariate analyses of EORTC-QLQ-BR23

	Symptomatic subscale			
	Side effects of systemic therapy OR (SE), ^*p*^ value	Arm symptoms OR (SE), ^*p*^ value	Breast symptoms OR (SE), ^*p*^ value	Hair loss OR (SE), ^*p*^ value
Disease free period				
(3rd year/1st year)		8.56 (1.69), <0.001	6.80 (1.32), <0.001	
(2nd year/1st year)		5.82 (1.45), <0.001	4.66 (1.16), <0.001	
Disease stage				
(III-IV/0)		-4.70 (2.29), 0.041	−4.21 (1.80), 0.019	
Axillary dissection				
(axillary/none)	−3.47 (0.98), <0.001	−4.83 (1.50), 0.001	−2.95 (1.17), 0.12	−8.72 (2.70), 0.001
Hormone therapy				
(yes/no)	−1.78 (0.56) 0.001			
Employment change				
(employed/employed➔unemployed)		2.76 (1.34),		
(unemployed/employed➔unemployed)		0.039	2.48 (1.24), 0.046	
Educational status				
(high/low or middle)		3.54 (1.43), 0.013		
Household income				
(low/high)	−4.23 (0.97), <0.001	−3.33 (1.40), 0.018	−2.12 (1.02), 0.038	
Hobbies				
(active/rare)	4.98 (1.14), <0.001	6.69 (1.58), <0.001	2.63 (1.24), 0.033	8.85 (3.34), 0.009
Medication of psychotropic drug	-			
(yes/no)	9.90 (1.85), <0.001	−7.09 (2.49), 0.004		
Medication of diabetes				
(yes/no)		−5.61 (2.52), 0.026		
Current smoking				
(yes/no)	−7.06 (2.99), 0.018			
Supplementary drug				
(yes/no)	−3.17 (0.95), 0.001			
	Functional subscale			
	Body Image OR (SE), ^*p*^ value	Future perspective OR (SE), ^*p*^ value	Sexual activity OR (SE), ^*p*^ value	Sexual Enjoyment OR (SE), ^*p*^ value
Disease free period				
(3rd year/1st year)	3.92 (1.83), 0.032			
Breast surgery				
(MRM/BCS)	-9.16 (2.82), 0.001			
Axillary dissection				
(axillary/none)				−5.55 (2.56), 0.031
Chemotherapy				
(yes/no)	−3.40 (0.78), <0.001		−1.15 (0.48), 0.018	
Hormone therapy				
(yes/no)				2.80 (1.32), 0.034
Radiotherapy field				
(breast/including regional nodes)				11.22 (2.98), <0.001
Menopausal status				
(post−/pre-)	4.88 (1.55), 0.002	6.65 (2.00), 0.001		
Age group				
(>60/<40)			−9.78 (1.49), <0.001	−38.16 (4.91), 0.001
(51–60/<40)			−4.22 (1.00), <0.001	−22.82 (4.11), <0.001
(41–50/<40)				−13.26 (4.01), 0.001
Employment change				
(employed/employed➔unemployed)	−8.74 (1.83), <0.001	−8.63 (2.31), <0.001		
Household income				
(low/high)	−3.61 (1.54), 0.019	−6.23 (1.98), 0.002		
Hobbies				
(active/rare)	6.16 (1.81), 0.001	9.38 (2.31), <0.001	6.27 (1.41), <0.001	6.67 (2.53), 0.009
(moderate/rare)			5.08 (1.16), <0.001	
Medication of psychotropic drug				
(yes/no)	−11.59 (2.85), <0.001			
Alcohol consumption				
(yes/no)	−3.82 (1.77), 0.031			
Supplementary drug				
(yes/no)	−4.34 (1.50), 0.004	−4.68 (1.95), 0.017		

Negative sign is a negative effect and positive sign is a positive effect

*Abbreviations*: *EORTC-QLQ-BR23*, the EORTC Quality of Life Questionnaire–breast cancer specific module, *OR* odds ratio, *SE* standard error, *MRM* modified radical mastectomy, *BCS* breast conserving surgery

## Discussion

This study presented the general index of QoL with statistical power and showed the change in QoL in the critical early phase after adjuvant radiotherapy. We were able to observe the difference in QoL pattern with time during the first 3 years after breast cancer treatment. Our data of EQ-5D-3 L showed that pain/discomfort and anxiety/depression were the most deteriorated categories. Regarding EORTC QLQ-BR23, hair loss in the symptom area and sexual activity in the function area were the most unsatisfactory categories in each area, for all surveillance periods.

Although there is little previous data investigating the priority between categories of EQ-5D or EORTC QLQ-BR23, our study showed similar results with other studies. Kim et al. performed a study with breast cancer patients after treatment, measuring QoL with instruments including EQ-5D. Between 5 categories of EQ-5D, far more patients answered with higher grades in categories of pain/discomfort and anxiety/depression [11]. In other studies that used EORTC QLQ-BR23 with breast cancer patients who completed treatment including radiotherapy, the upset by hair loss category had the lowest score in the symptom area [4]. Referring to another Korean study assessing QoL of breast cancer, sexual activity was the far lowest scored category in the functional area [15].

Several studies have reported that breast cancer survivors showed improvement over time in many domains of QoL including pain, breast and arm symptoms [16, 17]. The authors of a 10 year long-term follow up study reported that the symptoms of pain improved 3 years after treatment but aggravated 5 to 10 years after treatment [8]. In a recent study with early breast cancer patients who completed treatment including radiotherapy, the breast symptoms category of EORTC QLQ-BR23 was improved at 6–8 months and 2 years after treatment [18]. A French prospective study using EORTC QLQ-BR23 also showed that breast symptoms and arm symptom categories were significantly improved 3 and 6 months after surgery [19]. Our present study showed corroborated results that breast symptoms and arm symptom categories of EORTC BR-23 were significantly improved with the lapse of time. Improvement in EQ-5D VAS categorical data of pain/discomfort for the first 3 years after treatment is similar with the above mentioned study [8, 16, 17].

In out study, anxiety/depression category in EQ-5D VAS and future perspective category in EORTC QLQ-BR23, which reflect mental aspect, were not significantly improved. Psychosocial support may be needed considering their mental burden. Another point is that the categories of sexual activity and enjoyment in EORTC QLQ-BR23 scored far lower than other categories. We assume that the result is owing to atmosphere affected by Confucianism culture, where it is uncommon and difficult to discuss the sexual subject.

Various clinical and sociodemographic factors were investigated in our study. Multivariate analysis revealed that the axillary dissection was a prominent clinical factor affecting the largest number of QoL categories. Axillary dissection is the factor that might cause lymphedema related arm symptoms or other functional side effect, [20] hence postoperative rehabilitation is recommended and an effort to spare aggressive dissection should be continued. Many sociodemographic factors affected several categories of QoL in multivariate analysis; household income, activeness of hobbies, educational status, and occupational status were prominent. Other investigators also mentioned that socioeconomic factors were important determinants of QoL, including educational status, household income, and occupational factors [21, 22]. Some sociodemographic factors are difficult to change after cancer treatment; however, physical activity and activeness of hobbies could be boosted by various social programs. Previous studies using exercise intervention reported the positive impact on QoL such as depression, body image and sleep quality [23, 24].

The limitation of this study is that the data at each year was not derived from same patients, so we can only compare the data of population in relevant periods. Despite this disadvantage, this study is meaningful with investigation for a large number of patients in a multicenter group, and comprehensive categorical analysis of time-related QoL changes.

## Conclusion

Pain/discomfort and self-care categories in EQ-5D VAS, arm/breast symptoms and body image in QLQ-BR23 were improved during follow-up. On the other hand, Anxiety/depression in EQ-5D VAS and future perspective QLQ-BR23 has not improved, suggesting necessity of psychosocial support. Axillary dissection affected many categories of QoL and should be spared if possible, and various sociodemographic factors affect QoL, not only clinical factors. These results may be useful for follow-up consultation after treatment of breast cancer patients.

## Abbreviations

3 L: 3-Grade likert scale
EQ-5D: The 5-dimensional questionnaire by the EuroQol group
GPAQ: Global physical activity question
MVA: Multivariate analysis
QLQ-BR23: The EORTC Quality of Life Questionnaire-breast cancer specific module
QoL: Quality of life
VAS: Visual-analogue scale
WHO: World Health Organization
